# Potential Noninvasive Biomarkers to Assess the Aging Process

**DOI:** 10.1017/erm.2025.10020

**Published:** 2025-09-15

**Authors:** Álvaro Pérez Muñoz, Alejandro Gonzalez-Serna, Mercedes Cano

**Affiliations:** Department of Physiology, Faculty of Pharmacy, University of Seville, Seville, Spain

**Keywords:** aging, biomarkers, microbiota, senescence, telomere shortening

## Abstract

Aging is a process preserved in all living beings, progressive over time and inexorable. Despite the existence of several theories that attempt to explain changes associated with aging, scientists have not managed to satisfactorily explain the causes of aging. However, during the last decade, several cellular processes involved in the aging process have been shown to be involved, allowing scientists to identify new biomolecules as aging biomarkers and control the progression of aging. Currently, there is no single biomarker sensitive and specific enough to predict aging, so it is necessary to find a set of specific biomarkers of cellular processes involved in aging. These biomarkers must be accessible for quantification in biological samples in a noninvasive way to implement them in clinical practice. By 2050, it is estimated that approximately one in six people in the world will be over 65 years old, doubling the percentage of population over 60 years old. Therefore, the research of new biomarkers represents a novel strategy to counteract against aging and improve quality of life. In this review we summarize the potential biomarkers of aging that could be used in a noninvasive manner.

## Introduction

**Figure 1.**
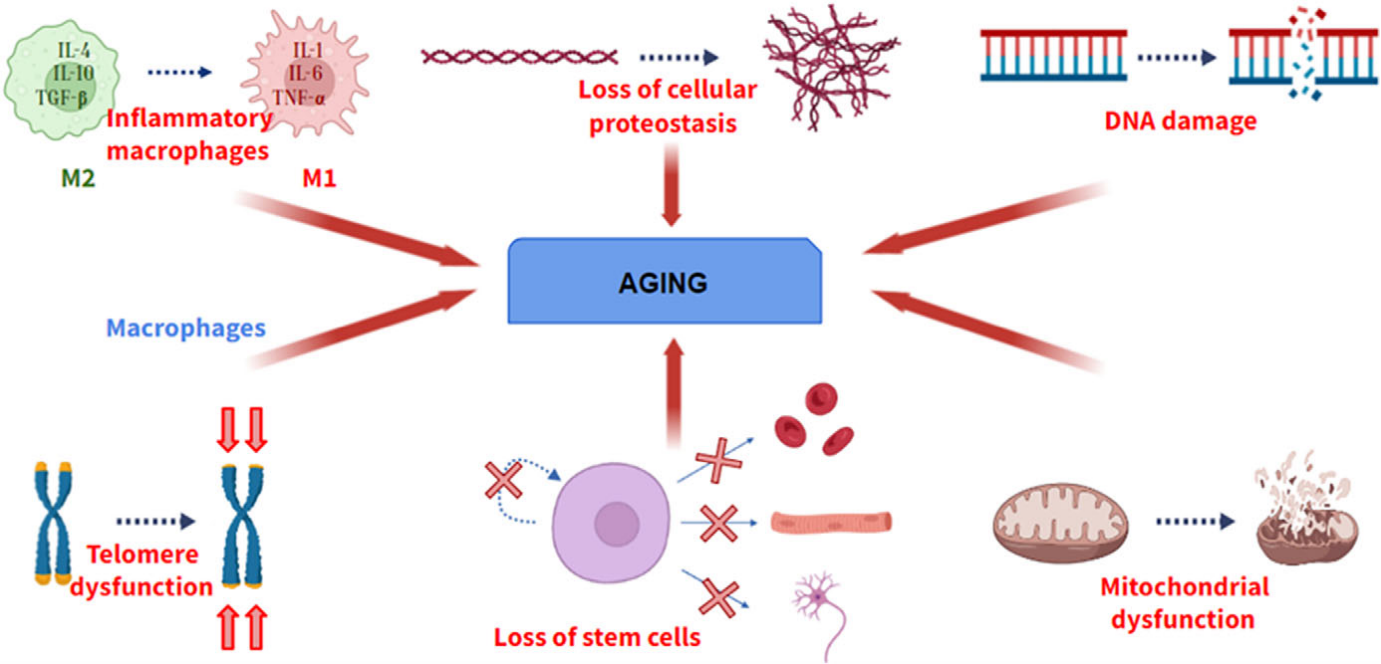
Some of the hallmarks of aging. From left to right: Change from M2 (anti-inflammatory) to M1 (pro-inflammatory) macrophage phenotype, loss of cellular proteostasis, increase in DNA damage, loss of telomere length, loss of differentiation and self-renewal of stem cells and mitochondrial dysfunction.

Aging is defined as a universal physiological process that involves the progressive loss of optimal functions of an organism. Aging is a multifactorial process, not due to a single cause. Although the causes of aging have not been elucidated, during the last decade, several researchers have shown several physiological processes that become deregulated during aging, providing valuable information about cellular and molecular pathways involved in aging. A hallmark of aging must meet three fundamental requirements: its manifestation must be associated with the age of the individual, its promotion experimentally must induce the acceleration of aging, and its inhibition experimentally must delay the progression of aging (Ref. 1). If a molecular process does not meet these characteristics, it cannot be considered a hallmark of aging. Among these processes (Figure 1), there are genomic instability, telomere shortening, the presence of certain epigenetic alterations, loss of cellular proteostasis, mitochondrial dysfunction and loss of stem cell functionality.

More than 300 theories have been proposed to explain aging. However, none of them has managed to explain the causes of aging. These theories can be divided into 2 groups: nonstochastic or deterministic theories and stochastic or environmental theories (Ref. 2). Deterministic theories postulate that aging is a process that is programmed by genetics and, therefore, is intrinsic to each individual, while the latter propose that aging is a process that can be modulated by environmental factors. An example of a deterministic theory would be Hayflick’s mitotic limit theory, which postulates that the number of cell divisions is programmed and once this limit is exceeded, the cell goes into senescence (Ref. 3). It is currently known that the number of divisions is determined by telomeres, noncoding DNA sequences located at the ends of chromosomes. Among the stochastic theories are catastrophic error theory, free radical theory, advanced glycosylation products theory and so forth The catastrophic error theory proposes that aging is a consequence of an error in protein synthesis, generating nonfunctional proteins that would interfere with DNA replication, increasing the number of mutations in the cell, thus leading to aging. The free radical’s theory is traditionally the most accepted by the scientific community, whose main defender was Denham Harman (Ref. 4). This theory postulates that free radicals, generated as a consequence of endogenous metabolism, exert harmful effects on biomolecules and therefore on the organism.

However, in recent years, the validity of this theory has been questioned, since it is currently known that free radicals at physiological concentrations are necessary to maintain cellular and tissue homeostasis. Because of that, scientists have proposed the free theory of fragility, which proposes that free radicals in low concentrations act as inducers of antioxidant enzymes and promote the adaptation of the human body to a stressful situation, such as exercise, while once they exceed a threshold concentration, antioxidant enzymes cannot eliminate them and oxidative damage occurs (Ref. 5). Therefore, this theory proposes that free radicals act through a mechanism of hormesis in the aging process.

**Figure 2.**
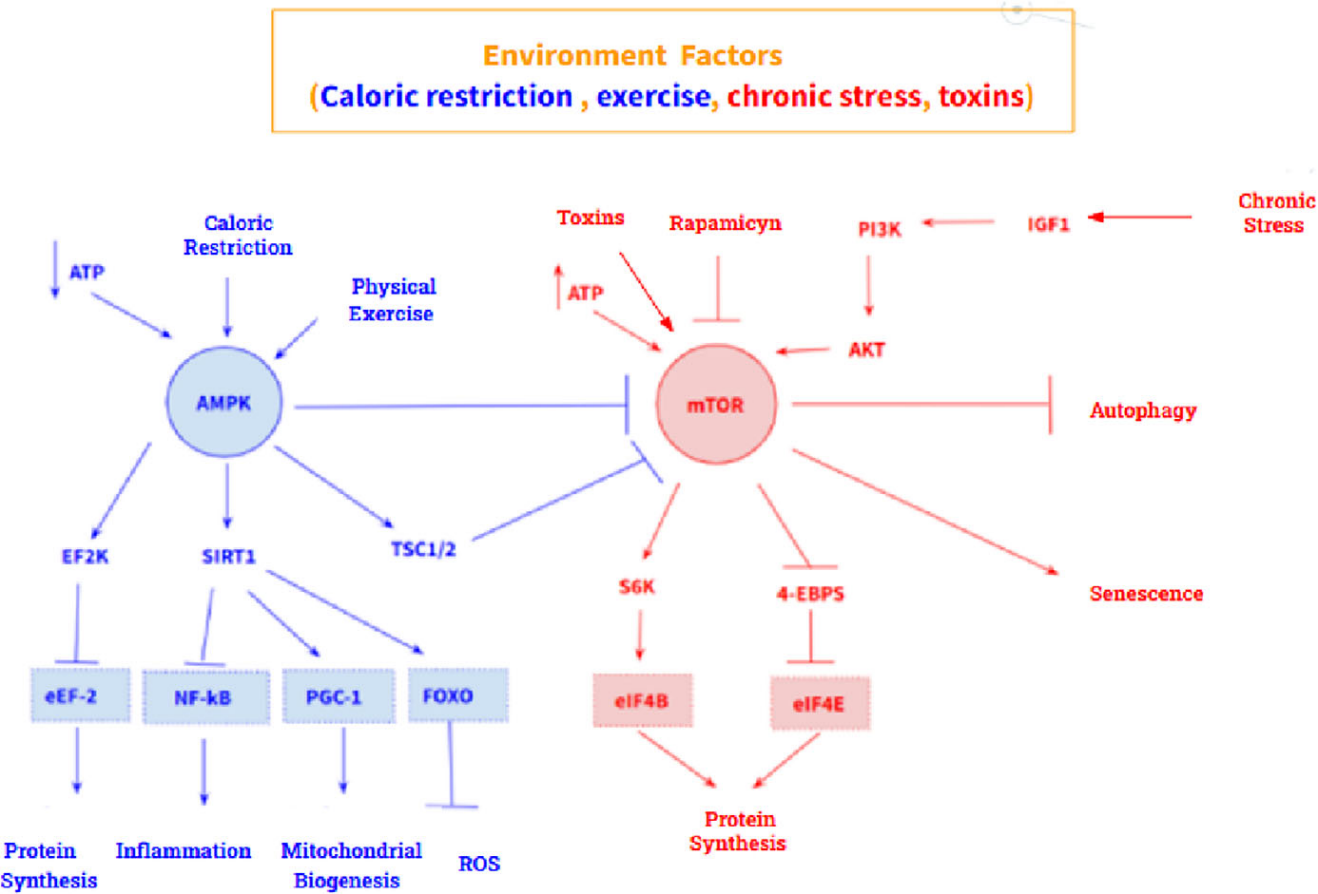
AMPK-mediated (blue) and MTOR-mediated (red) anti-aging signalling pathway. Both routes are highly modulable by the environment.

In addition, two molecular pathways stand out in aging: an antiaging pathway and a pro-aging pathway. The anti-aging pathway is mediated by the AMP – activated protein kinase (AMPK), a sensor of intracellular ATP concentration, which is activated by a low concentration of ATP. Its main target is mechanistic target of rapamycin kinase (MTOR), which it inhibits. Furthermore, AMPK also acts on eukaryotic elongation factor 2 kinase (EEF2K) by activating it, which causes inhibition of eukaryotic translation elongation factor 2 (EEF2), thus promoting inhibition of protein synthesis. On the other hand, the pro-aging pathway is mediated by MTOR, whose main targets are the ribosomal protein S6 kinase (RPS6KA1) and the eukaryotic translation initiation factor 4E binding protein 1 (EIF4EBP1) whose activation triggers protein synthesis and cell growth (Figure 2). However, these two pathways are highly influenced by the environment, so their expression is modulable. Calorie restriction and physical activity promote AMPK activation, while excess nutrients, as well as certain growth factors such as insulin like growth factor 1 (IGF1), activate MTOR. At the pharmacological level, metformin and rapamycin stand out as drugs that modulate both pathways: metformin is an activator of AMPK, and rapamycin is an inhibitor of MTOR (Ref. 6).

### Biomarkers in aging

A biomarker is a characteristic that can be measured and evaluated as an indicator of a physiological or pathological event. Related to aging, the first formal definition of an aging biomarker was proposed in 1988: ‘biological parameter of an organism that in an individual analysis or in combination with other parameters, in the absence of disease, allows one to estimate the functional capacity of an organism over a longer term than its chronological age’ (Ref. 7). According to the AFAR (American Federation for Aging Research), a good aging biomarker must be a parameter that meets the following characteristics (Ref. 8): (1) It must predict biological age, evaluating the physical, cognitive or physiological capacity of an individual, independently of their chronological age; (2) It must be measurable without difficulty, that is, it can be measured routinely and without causing harm to the person, for example, through a blood test; (3) It must monitor a biochemical process linked to aging, that is, a *hallmark of aging*, whose manifestation is not a consequence of any pathology, but of aging itself; (4) It must be able to be monitored in experimental models (especially in mice), so that animal–human extrapolations can be made.

Regarding the search for biomarkers of aging, blood has been proposed as the best tissue in the human body for the search for biomarkers that inform the aging process, since the existence of certain proteins whose concentration fluctuates with age has been discovered (Ref. 9). Among the experimental evidence that supports this hypothesis are the parabiosis experiments, through which it has been shown that the blood of a young mouse, if transfused into an old one, rejuvenates its tissues and vice versa (Ref. 10). If blood is transfused from the old animal to the young, the young animal will suffer accelerated aging. This confirms that in the blood of the young animal, there must be some soluble factor that induces the old tissues to rejuvenate, just as in the blood of the old animal, there must be some molecule that causes the young animal to undergo accelerated aging.

## Immune system markers

During aging, different changes occur in the immune system that are included in the biological process known as immunosenescence. On the one hand, innate immunity changes with aging, which constitutes the first line of defence against pathogens and lacks specificity. Among its components of innate immunity are dendritic cells, the monocyte–macrophage system, neutrophils and NK lymphocytes. NK lymphocytes are the most studied during the aging process.

NK cells are natural killer lymphocytes that originate in the bone marrow of the lymphoid precursor, which have innate receptors that detect the absence of molecules of the major histocompatibility complex class I (MHC-I), which occur mainly in virus-infected cells and tumour cells. They also express the receptor for low-affinity IgG (FCGR3A) and the CD56 molecule (Ref. 11). Most studies show a slight reduction in the number of NK lymphocytes as aging progresses, which are determined by flow cytometry as CD45^+^, CD56^+^ and CD3^−^, as well as a considerable decrease in their cytotoxic activity. This activity can be evaluated *in vitro* through multiparametric flow cytometry-based cytotoxicity assays to determine the expression of the LAMP1 protein, a major and highly glycosylated transmembrane protein located in the lysosomes of these cells. This technique is based on the process of fusion of cytolytic granules with the plasma membrane just when recognition of the target cell by the NK cell takes place, a process known as degranulation. Once the fusion of the granules with the plasma membrane occurs, the translocation of LAMP1 to the membrane occurs, so the absence of its expression is indicative of absent or decreased cytolytic activity (Ref. 12). In addition to the decrease in their cytolytic activity, during aging there is an increase in the percentage of CD56 ^dim^ NK lymphocytes and a decrease in the percentage of CD56^bright^ NK lymphocytes (Ref. 13). On the other hand, studies that have conducted single-cell RNA sequencing have identified a decrease in the levels of the cytokines *MIP1a, RANTES, CXCL8, IL2, IFNG, TNFA* and *IL12* during aging, thus contributing to the poor functioning of the immune system (Ref. 14). These cytokines can be quantified in serum as a biomarker of aging using the ELISA technique.

T lymphocytes and B lymphocytes are the cellular elements involved in adaptive immunity. Adaptive immunity differs from the innate response in strategy and in pathogen recognition mechanisms, since T and B lymphocytes specifically recognize particular structures present in pathogens called antigens through the T-cell receptor (TCR) and B-cell receptor (BCR) respectively. Among the changes that occur during adaptive immunity aging, the decrease in the CD4/CD8 T lymphocyte ratio is the main event, which means the reduction in the number of helper CD4 T lymphocytes. Furthermore, it should be noted that the inversion of this ratio is more pronounced in human immunodeficiency virus (HIV)-positive patients, thus establishing a connection between the pathogenic mechanisms of infection and the aging process (Ref. 15). Furthermore, changes will also occur in CD8 T lymphocytes, fundamentally a decrease in both their proliferation and their cytotoxic activity, which leads to a greater susceptibility to infections and tumours.

Regarding CD8 T lymphocytes, flow cytometry is the main method used to study them. This technique analyses certain surface molecules, including PTPRC, CD27, SELL and CCR7. The markers PTPRC and B3GAT1 are also measured, which are indicative of cellular senescence and their expression increases during aging. The β-galactosidase activity can also be tested in these cells, indicating the level of cellular senescence (Ref. 16). During aging, β-galactosidase activity is high in CD8 T lymphocytes and is associated with telomeric disfunction and transcript levels of the senescence marker p16 (Ref. 17).

Aging leads to a decline in B lymphocytes, which are essential for the humoral immune response, driven by multiple biological mechanisms: first, IL7 synthesis decreases during aging, a cytokine necessary for the survival and proliferation of B lymphocyte precursors, preventing the generation of new B lymphocytes. Second, the expression of the transcription factors E2A and paired box protein 5 (PAX5) decreases during aging, which are necessary for differentiation of the B-cell progenitor. Furthermore, different studies have described the accumulation of a new cell type, age-associated B cells (ABCs) during aging. These cells show a high capacity to secrete TNFA (tumour necrosis factor alpha) which inhibits the generation of new pro-B lymphocytes and therefore functional B lymphocytes. Additionally, TNFA synthesis contributes to the promotion of chronic inflammation characteristic of aging, accelerating its course (Ref. 18).

The following table (Table 1) summarizes the main changes that occur in the immune system during aging, which can be used to evaluate its progress in healthy people and those with pathologies. Among these changes, measurement of pro-inflammatory cytokines in plasma using ELISA, such as IL1B, IL6 and TNFA, is useful for studying chronic inflammation during the aging process and can be applied in clinical practice (Ref. 19).

**Table 1.** Summary of changes in the immune system during aging

Parameter	Successful aging (centenarians)	Aging pathological	References
CD4^+^ T lymphocytes	↑ Expansion	↑ Th1 ↑ Th17	(Ref. 15)
CD8^+^ T lymphocytes	↑ Proliferation ↑ Cytotoxicity	↓ Proliferation ↓ Cytotoxicity	(Ref. 18)
B lymphocytes	↑ Naive B cells ↓ ABCs	↓ Proliferation ↑ ABCs	(Ref. 18)
NK lymphocytes	↑ Cytotoxicity ↑ IFNG	↑ CD56 ^dim^ ↓ CD56 ^bright^	(Ref. 13)
Inflammatory profile	↑ TGFB ↑ IL10 ↑ IL1RA	↑ IL1B ↑ IL6 ↑ TNFA ↑ CRP	(Ref. 19)

TGF-β: Transforming Growth Factor-beta; IL10: Interleukin-10; IL1R1: Interleukin-1 Receptor Antagonist ; IL1B: Interleukin-1 beta; IL6: Interleukin-6; TNFA: Tumour Necrosis Factor-alpha; CRP: C-Reactive Protein; ABCs: Aged B Cells; Th1: T-helper 1; Th17: T-helper 17.

*Note*: Table adapted from Wang et al. (Ref. 16).

On the other hand, the decrease in telomerase activity in lymphocytes produced during aging leads to a lower lymphoproliferative response to pathogens, causing poor elimination of them and leading to greater susceptibility to infections (Ref. 16). Therefore, measurement of the length of the telomeres in lymphocytes constitutes an interesting marker for studying the speed of the aging process. To carry out this measurement, real-time quantitative PCR (qPCR) is mainly used, which allows the relative quantification of the length of the telomere by calculating the T/S ratio. The T/S ratio is an experimental quantification commonly used to calculate telomere length. Two parameters are necessary for estimating it: telomeric DNA (T) and single copy gene (S). It should be noted that qPCR permits measurement with small amounts of DNA, so greater variability of biological samples can be used, such as blood, cells from the oral cavity, etc.

## Senescent cells

**Figure 3.**
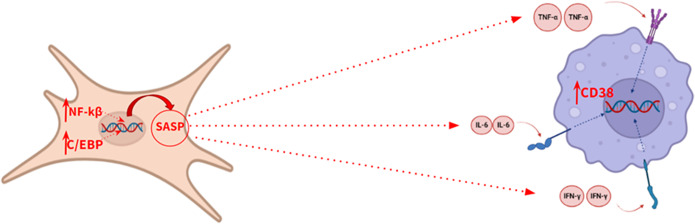
Illustrative model of the secretory phenotype associated with senescence.

Senescent cells play a prominent role in the pathophysiology of aging. These cells are mainly characterized by the expression of the enzyme SA-β-galactosidase and p16, a tumour suppressor protein encoded by the *CDKN2A* gene, thus allowing their identification (Ref. 20). However, the secretory associated senescence phenotype (SASP) is the most relevant biochemical feature of these cells. The activation of this phenotype is mediated by the action of the C/EBP and NFKB transcription factors. Biochemically, this phenotype is characterized by the release of pro-inflammatory cytokines, such as TNFA, IL6, CXCL8, IFNG, CCL2 and IL1B, which promote a state of chronic inflammation (Ref. 19), accelerating the aging process (Figure 3), so the quantification of these cytokines is useful for monitoring the progress of aging. This biological process has also been described at the brain level, where senescent astrocytes release pro-inflammatory cytokines in a paracrine manner, causing premature aging of microglia (Ref. 21).

Furthermore, under the influence of these cytokines, an increased expression of CD38 has been shown in tissues adjacent to senescent cells. CD38 is a transmembrane glycoprotein with enzyme activity that degrades the nicotinamide adenine dinucleotide (NAD^+^) metabolite through its glycohydrolase activity (Ref. 21), which affects the biological activity of sirtuins, altering the maintenance of cell homeostasis, thus accelerating aging. Sirtuins are histone deacetylase enzymes which regulate signalling pathways in eukaryotes, playing a variety of significant roles in cellular physiology, such as inflammation, oxidative stress or apoptosis. Therefore, an increase in CD38 expression would accelerate the aging process by reducing the level of NAD^+^ and, therefore, decreasing sirtuin activity. This makes CD38 protein an interesting biomarker of aging. According to several authors, spectrophotometry is the most suitable method in order to measure CD38 hydrolase activity in any tissue (Ref. 22).

## Oxidative stress markers

Oxidative stress represents one of the key biological mechanisms driving both the aging process and its associated pathologies, particularly neurodegenerative diseases including Alzheimer’s disease (AD), Parkinson’s disease (PD) and Amyotrophic Lateral Sclerosis (ALS). This oxidative stress is produced in response to a situation of imbalance between the endogenous synthesis of reactive oxygen species (ROS) and the action of antioxidant defences, the chronification of this situation is a promoter of the aging process (Ref. 23). Therefore, biomarkers that report on individual oxidative stress and can be measured in plasma must also report on aging progress.

Regarding the biomarkers most widely used to measure oxidative stress, the enzymes involved in the defence of cellular antioxidants are the most important, among them superoxide dismutase (SOD), catalase (CAT) and glutathione peroxidase (GPx) (Table 2). SOD catalyses the dismutation reaction of the superoxide anion into oxygen and hydrogen peroxide, and its mutation is responsible for 1.2% of cases of sporadic amyotrophic lateral sclerosis (sALS) and 14.8% of cases of familial ALS (fALS), demonstrating the importance of its biological function (Ref. 24). In turn, hydrogen peroxide is highly toxic, so it is metabolized by CAT, which transforms it into water and molecular oxygen. Therefore, both enzymes are key to controlling redox balance and neurodegeneration (Ref. 25), which makes them interesting biomarkers for studying aging.

**Table 2.** Main biomarkers of oxidative stress

Molecular parameter	Biological function	Biological Sample	Normal values	Sex difference	Levels inAging	References
Superoxide dismutase (SOD)	Antioxidant defence	Plasma	6.10–7.44 U/ mg prot	♂ < ♀	Decreased	(Ref. 49)
Glutathione peroxidase (GPx)	Antioxidant defence	Plasma	27.5–73.6 U/g of Hb	♂ < ♀	Decreased	(Ref. 49)
Malondialdehyde (MDA)	Product of lipid peroxidation	Plasma	< 2.4 μmol/L	♂ < ♀	Increased	(Ref. 49)
GSH/GSSG	Antioxidant defence	PBMCs	>100	♂ > ♀	Decreased	(Ref. 49)
Vitamin E	Lipid antioxidant	Plasma	4.9–19.9 mg/mL	♂ > ♀	Decreased	(Ref. 29)

PBMCS: Peripheral blood mononuclear cell; SOD: Superoxide dismutase; GPX: Glutathione peroxidase; MDA: Malondialdehyde; GSH: Reduced glutathione; GSSG: Oxidized glutathione.

*Note*: Adapted from Ref. 42.

**Figure 4.**
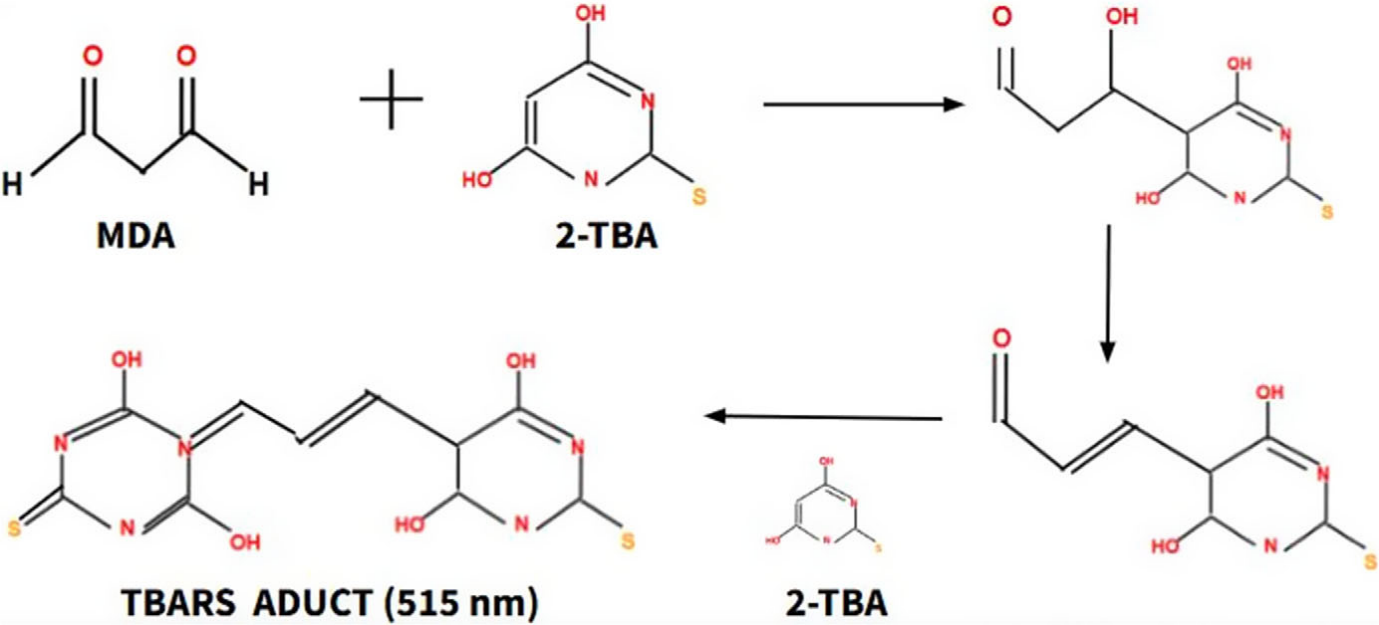
The formation process of the TBARS adduct, a product of the reaction of MDA with TBA, allows the concentration of MDA to be indirectly calculated. Thiobarbituric acid reactive substances (TBARS), Malondialdehyde (MDA), Thiobarbituric acid (TBA).

On the other hand, lipid peroxidation processes, which are involved in damage to the plasma membrane and therefore in the progression of aging, are defined as a process of oxidative damage to lipids mediated by ROS (Ref. 26). There are several molecular biomarkers to monitor lipid peroxidation processes, such as malondialdehyde (MDA), 4-hydroxy-2,3-trans-nonenal (4-HNE) and isoprostanes. MDA is the most reliable biomarker, as its measurement is very easy in plasma. High-performance liquid chromatography (HPLC) is the gold standard technique, which takes advantage of MDA reactivity capacity with 2-thiobarbituric acid (TBA), forming an adduct that can absorb light at a wavelength of 515 nm, this absorption is proportional to the concentration of MDA (Figure 4). Different studies have shown an increase in MDA concentration as aging progresses and have shown that the value indicative of normality in the blood is less than 2.4 mmol /L (Ref. 27).

Moreover, it is worth mentioning the role of vitamin E during aging, since different studies have shown antioxidant and anti-inflammatory benefits. Vitamin E can inhibit cyclooxygenase 2 (PTGS2), thus decreasing the synthesis of prostaglandin E2 (PGE2), a pro-inflammatory mediator. Furthermore, it can slow down the synthesis of the pro-inflammatory cytokines TNFA, IL6 and IL1 allowing for the chronic inflammation associated with aging. Regarding its functions in the immune system, vitamin E plays an immunomodulatory role, as it promotes the Th1 response through the synthesis of IFNG, a cytokine with immunomodulatory activity (Ref. 28). Due to these reasons, measurement of vitamin E in plasma through HPLC is useful in order to assess the level of oxidative stress and chronic inflammation of an individual. The concentration of vitamin E in plasma oscillates between 12 and 20 mmol/L, whose levels decrease with age, thus predisposing to a greater risk of age-related chronic diseases, such as anaemia, infections or dementia (Ref. 29).

## Microbiota markers

The human microbiota is defined as the set of microorganisms that inhabit the digestive tract, with an estimated number between 10^13^ and 10^14^ microorganisms. There are two predominant phyla, Bacteroidetes and Firmicutes, which together constitute more than 90% of the human microbiota. The most abundant genera are *Bacteroides*, *Prevotella* and *Ruminococcus. Bacteroides and Prevotella* belong to the phylum Bacteroidetes, whereas *Ruminococcus* belongs to the phylum Firmicutes. It has been described that microbiota remains stable for decades from 3 years of age, although different studies have described a decrease in the population of these genera as aging progresses (Ref. 30). Exploring more in detail, it has been shown that with age, there is an increase in the *Proteobacteria phylum*, which includes a wide variety of pathogenic bacteria such as *Escherichia coli*, *Salmonella* and *Neisseria*, while some butyrate-producing bacteria decrease, including *Ruminococcus obeum*, *Eubacterium rectale* or *Faecalibacterium prausnitzii.* The latter is worth highlighting as it is the main butyrate-producing bacterium in the intestine, and different researchers have demonstrated its protective role against intestinal inflammation because of its immunomodulatory capacity. It exerts different mechanisms of action, for example, the stimulation of the synthesis of IL10 and regulatory T cells or the inhibition of NFKB (Ref. 31). Therefore, monitoring the presence of this bacterium can be useful to control aging.

**Figure 5.**
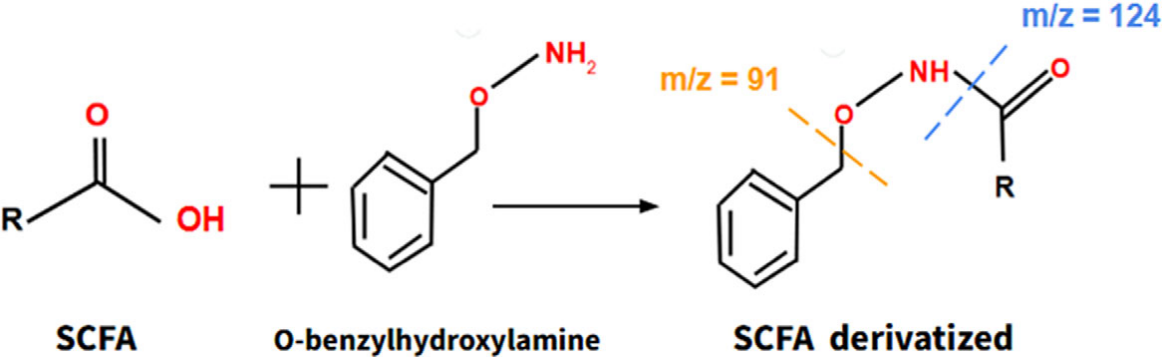
The formation process of derivatized SCFA, a product of the reaction of SCFA with O-benzylhydroxylamine. Derivatized SCFA can be assessed by LC–MS. Short Chain Fatty Acids (SCFA) Liquid Chromatography–Mass Spectrometry (LC–MS).

Moreover, it is important to consider the relationship between microbiota and the human physiology, as the microbiota synthesizes different beneficial compounds that promote the proper functioning of human metabolism, including short chain fatty acids (SCFAs). These SCFAs, once synthesized in the intestine, migrate to the bloodstream, where they act by promoting beneficial actions, such as reducing cholesterol and triglyceride levels, decreasing resistance to insulin and so forth As aging progresses, the microbiota–host relationship deteriorates, so SCFAs synthesized by the microbiota may be useful as indicators of this relationship and constitute suitable biomarkers of aging. Actually, several studies have shown that SCFAs can mitigate clinical manifestations of aging in mice, such as sarcopenia or cognitive impairment (Ref. 32). SCFAs can be obtained from a blood sample and they can be assessed through a derivatization reaction using O-benzylhydroxylamine as a derivatizing agent (Figure 5), combined with liquid chromatography and mass spectrometry (LC–MS) through detection of its main molecular ions according to the mass–charge (m/z) ratio (Ref. 33).

It is also necessary to highlight the bacteria *Akkermansia muciniphila*, a gram-negative bacteria belonging to the Verrucomicrobia phylum, since its absence in the intestine is associated with intestinal inflammation and activation of the immune response (Ref. 34). Furthermore, the presence of this bacteria in the intestinal tract has been shown to have immunomodulatory properties, increasing the synthesis of different anti-inflammatory metabolites at the intestinal level, including different polyamines of the spermidine family and some SCFAs such as propionate, acetate and butyrate. These metabolites are essential for maintaining intestinal permeability through the reinforcement of intercellular junctions and for delaying aging. Different studies have shown that treatment with these bacteria in mice shows anti-obesity and anti-diabetes effects, increasing the number of regulatory T cells and decreasing the synthesis of pro-inflammatory cytokines, such as TNFA and IL6 (Ref. 35). Therefore, given their special relevance in inflammatory processes, the study of the bacteria *F. prausnitzii* and *A. muciniphila* is key to the control of aging and associated diseases. These bacteria can be isolated from bacterial culture at 37°C in an anaerobic chamber containing 90% N_2_, 5% CO_2_ and 5% H_2_. However, according to several authors, 16S rRNA gene-based phylogenetic analyses, which is based on amplification and sequencing of the hypervariable region of 16S rRNA gene, is one of the best methods for detecting *F. prausnitzii* in patients collected samples due to its high sensitivity and specificity (Ref. 36). In addition, *A. muciniphila* can also be detected by these metagenomic methods.

## Markers of genomic instability and DNA damage

Genomic instability is defined as the loss of efficiency of DNA repair mechanisms that cause DNA damage to increase and perpetuate over time. H2A.X variant histone is one of the most studied markers that indicate DNA damage, encoded by the *H2AX* gene. This protein is phosphorylated at serine residue 139 in response to a double-strand break, thus being called gH2AX (S139), whose levels are proportional to the magnitude of DNA damage (Ref. 37). Some studies have shown a relationship between high levels of gH2AX (S139) and individuals with higher levels of frailty according to Linda Fried’s criteria (Ref. 38), thus suggesting that gH2AX (S139) is not only a simple indicator of DNA damage, but its quantification through Western blot can be used as a tool to control aging and determine, from a certain age, people who have a greater probability of evolving towards a state of fragility and pathological aging.

Plasma cell-free DNA analysis (cfDNA, circulating DNA) in samples of lymphoblasts/lymphocytes from patients is another interesting biomarker of aging. Different studies have shown a correlation between the levels in blood and the degree of fragility, according to Linda Freid’s criteria (Ref. 38). In fact, cfDNA levels increase with age, reflecting a higher rate of cell death and turnover in aging tissues. Older individuals tend to have higher cfDNA concentrations in their blood, potentially due to increased apoptosis (programmed cell death) and necrosis.

Furthermore, 8-hydroxy-2′-deoxyguanosine (8-OhdG) is a biomarker that reflects the level of oxidative damage at the nitrogenous bases of DNA. Measurements are usually carried out in urine and LC and gas chromatography are the most widely used analytical methods due to their high sensitivity and specificity. However, immunological techniques such as ELISA are gaining significant importance, as they are cheaper and faster than other analytical methods. Regarding reference values, different epidemiological studies carried out in the Italian population establish a range between 3.25 and 6.85 ng 8-OHdG/mg creatinine for women, while for men this range oscillates between 2.9 and 5.5 ng 8-OHdG/mg creatinine (Ref. 39).

## Epigenetic markers

Epigenetics refers to heritable modifications in gene expression that occur without changes to the DNA sequence itself. These modifications are highly sensitive to environmental influences (Ref. 40). Among the different epigenetic alterations that occur during aging, DNA methylation is the best known. It has been shown that the DNA methylation pattern undergoes modifications as time progresses; thus, alterations in these patterns must reflect the biological age of an individual. Because of that, different biomedical tools have been developed, called ‘epigenetic clocks’. These epigenetic clocks assess methylation patterns across CpG islands, which are DNA segments containing repetitive cytosine–guanine (CG) nucleotide pairs.

Currently, four epigenetic clocks stand out: horvath epigenetic clock, Hannun epigenetic clock, PhenoAgeDNAm and GrimAge DNAm. Each epigenetic clock selects certain CpG islands (Ref. 41). The Horvath and Hannum epigenetic clocks represent the first generation of epigenetic clocks, the Horvath clock being the first to be developed. The Horvath clock is based on the study of the methylation status of 353 CpG islands, 193 correlating positively with biological age and 160 correlating negatively. Once the methylation status of these islands has been elucidated, a mathematical algorithm predicts, through a penalized regression model, the biological age based on the methylation pattern found (Ref. 42). Hannun and Horvath epigenetic clocks use the same mathematical model to calculate biological age although they show an important difference: the Horvath clock allows scientists to estimate the biological age of different human tissues, while the Hannun clock only allows one to calculate the biological age through a blood sample, limiting its potential (Ref. 43). In addition, the DNAm PhenoAge epigenetic clock, as well as the study of the methylation pattern, is able to accurately elucidate the biological age in a clinical context. This epigenetic clock, as well as studying the methylation pattern of 513 CpG islands, uses 10 clinical biomarkers, which in combination with the methylation pattern, allow for a more precise estimate of biological age in a clinical context. These biomarkers are chronological age, albumin, creatinine, glucose, C-reactive protein, lymphocyte percentage, mean corpuscular volume, erythrocyte distribution width, alkaline phosphatase and white blood cell count (Ref. 44). The main advantages of DNAm PhenoAge compared to the Harvath and Hannum clock are that it allows scientists and doctors to know which patients are at risk of suffering from age-related diseases, making it possible to carry out preventive interventions, such as changes in lifestyle or pharmacological therapies that delay aging and therefore prevent age-related diseases (Ref. 44).

Finally, the DNAm GrimAge is based on the combination of different factors to estimate biological age. Firstly, the study of the methylation pattern of 1030 CpG islands, seven plasma proteins: cystatin C, leptin, plasminogen activator inhibitor 1, TIMP metallopeptidase inhibitor 1 (TIMP1), adrenomedullin, beta-2-microglobulin (B2M) and growth differentiation factor 15 (GDF15). All of these biomolecules are indicators of the cardiovascular, inflammatory and cognitive status of an individual. Secondly, it considers the years of smoking. The main advantage of this epigenetic clock is the incorporation of lifestyle habits and clinical biomarkers associated with age, allowing a more precise approximation of biological age and life expectancy, as well as predicting greater future risks in terms of possible pathologies (Ref. 45).

The following table summarizes the main characteristics of the four epigenetic clocks (Table 3), including the number of CpG islands studied; the type of biological sample, the mathematical model used, the correlation coefficient, an indicator of the level of reliability of the prediction and the year of publication.

**Table 3.** Characteristics of the main epigenetic clocks

Epigenetic clock	Horvath	Hannum	DNA mPheno Age	DNAm Grim Age
Number of islands CpGs	353	71	513	1030
Type of sample biological	Multiple fabrics	Blood	Blood	Blood
Mathematical model used	Regression penalized	Regression penalized	Penalized Cox Regression	Penalized Cox elastic Regression
Coefficient of correlation (r)	0.960	0.905	Not known	Not known
Year of elaboration	2013	2013	2018	2019
References	(Ref. 42)	(Ref. 43)	(Ref. 44)	(Ref. 45)

*Note:* Table adapted from Duan et al. (Ref. 41).

## Klotho protein as a biomarker of aging

**Figure 6.**
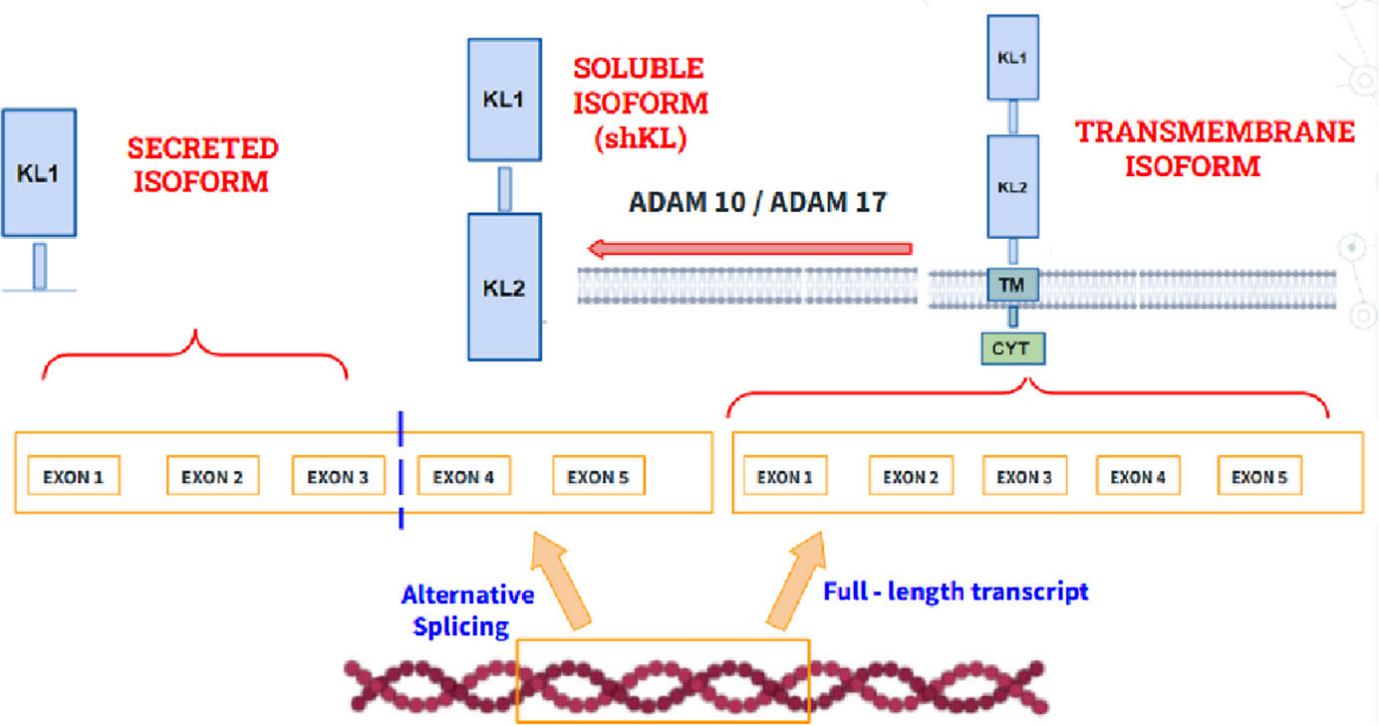
Structure of the transmembrane isoform of klotho (right), the secreted isoform (left), which is made by alternative splicing and the soluble isoform (shKL), product of the cleavage of the extracellular domains at the level of the transmembrane domain by the metalloproteinases ADAM10 and ADAM1A. Disintegrin and Metalloproteinase domain-containing protein 10 (ADAM10), A Disintegrin and Metalloproteinase domain-containing protein 17 (ADAM17), Klotho Transmembrane domain (TM), Klotho Cytoplasmatic domain (CYT), Klotho Extracellular domain 1 (KL1), Klotho Extracellular domain 2 (KL2).

The KL gene (encoding Klotho protein) maps to chromosome 13 is a type I transmembrane protein that plays a very important role during the aging process because of its anti-inflammatory and cognitive functions. Regarding its role in aging, different studies have shown that mutations in the *KL* gene cause premature aging with clinical manifestations such as osteoporosis or sarcopenia while its overexpression in mice increases life expectancy, thus demonstrating its potential to control and delay aging. Regarding its biochemical structure (Figure 6), three isoforms can be distinguished: (1) A transmembrane isoform. (2) A secreted isoform, which is made by splicing the mRNA gene that encodes it. (3) An isoform that circulates in blood, called shed KL (shKL), which is made by enzymatic cleavage of the transmembrane isoform. This isoform is the most abundant and predominates in blood, urine and cerebrospinal fluid (Ref. 46). Likewise, it has been well demonstrated that its endogenous synthesis decreases significantly during aging, so the quantification of its circulating isoform in blood may be promising to monitor its progression (Ref. 47).

**Figure 7.**
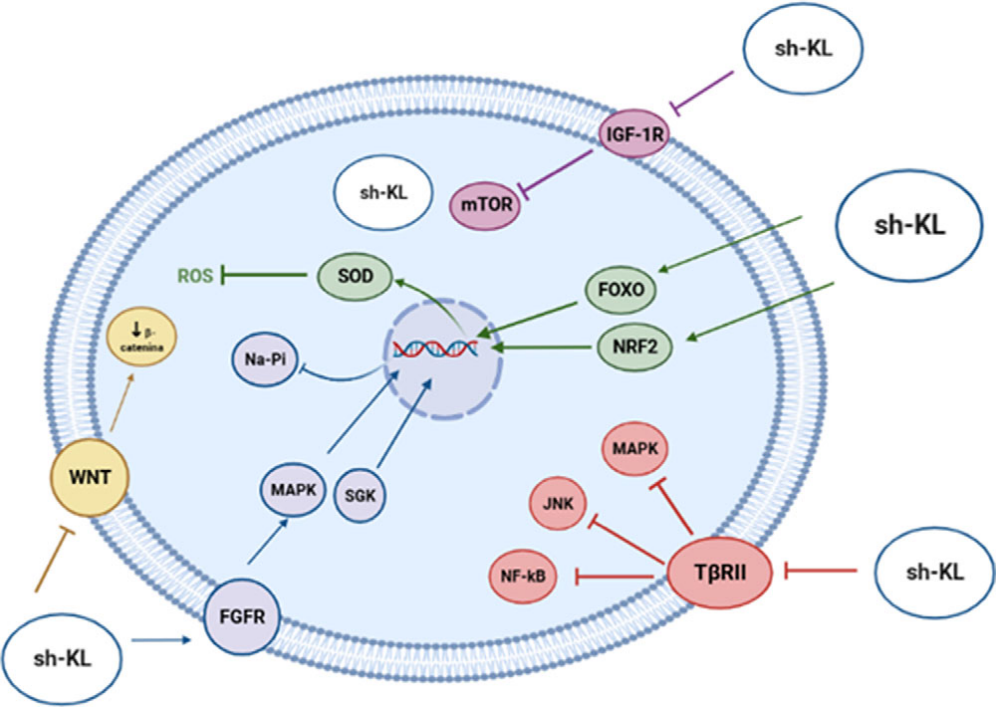
Biological functions played by Klotho on biochemical pathways that control aging.

As a soluble hormone, klotho modulates biochemical pathways that control aging and longevity through different mechanisms (Figure 7), such as the inhibition of IGF-1 receptor phosphorylation, the activation of antioxidant transcription factors and the transcriptional inhibition of genes which encode proinflammatory cytokines. Finally, klotho joins Wnt family member ligands, such as WNT1, WNT4 or WNT5A, thus blocking cellular senescence, tumorigenesis and fibrosis (Ref. 46).

Therefore, all the processes above mentioned prove the direct involvement of Klotho during aging, opening new lines of research for scientists to delay aging, for example, through its exogenous supplementation or through a drug which acts as a booster, promoting its endogenous biosynthesis.

On the other hand, it is a fact that the study of serum klotho levels has not yet been implemented in clinical laboratories, limiting clinical research by physicians. However, it is worth highlighting the research carried out during the first months of 2022 by the clinical analysis service of Virgen de las Nieves Universitary Hospital. During this research, a simple linear regression statistical analysis was performed to study the correlation between chronological age and serum klotho levels, showing a statistically significant negative association between both variables, as a p-value <0.01 was obtained, supporting the hypothesis that klotho levels decrease as chronological age increases, thus demonstrating that klotho levels are conditioned by the age and could be a good aging biomarker (Ref. 48).

## Glycans and glycanAge: novel predictive biomarkers in aging research

Glycosylation is a complex, epigenetically regulated process involving hundreds of genes and proteins. Age-related changes in glycosylation have been consistently observed and glycan-based biomarkers, particularly, those derived from immunoglobulin G (IgG) glycosylation, have shown strong associations with aging and systemic inflammation. Furthermore, alterations in IgG glycosylation can modulate its proinflammatory or anti-inflammatory activity, thus highlighting its relevance in the context of aging and immune function (Ref. 49). Delving deeper into this aspect, recent studies have shown that IgG sialylation is associated with AD and hypertension, whereas IgG fucosylation has been linked to multiple sclerosis. Additionally, monogalactosylation of IgG1 and IgG4 has been observed in patients with type 2 diabetes mellitus (T2DM). Moreover, patients with Down syndrome exhibit a distinct IgG glycosylation profile compared to healthy individuals, characterized by a higher abundance of agalactosylated and sialylated glycans, a pattern consistent with accelerated biological aging (Ref. 50).

## Proteostasis decline in aging: blood-based biomarker perspectives

Proteostasis is a cellular network for maintaining protein folding, function and degradation, playing a crucial role in preserving cellular homeostasis. During aging, this system progressively deteriorates, leading to the accumulation of misfolded and damaged proteins. The decline of this cellular process has been implicated in the pathogenesis of several age-related diseases, including neurodegenerative and metabolic disorders. Several clinical studies have shown elevated levels of 20S proteasome complex in the plasma of patients with aging-associated conditions, such as solid tumours and autoimmune diseases (Ref. 51).

## Pharmacotherapy of aging: drug-based interventions

Pharmacological modulation of the aging process through drug-based interventions has gained increasing interest in the scientific community in recent years. Several compounds – rapamycin, metformin, resveratrol and aspirin – have emerged as promising pharmacological agents with evidence demonstrating their capacity to delay aging and age-related diseases in animal models. Interestingly, many of these compounds have also been shown to modulate the composition and function of the gut microbiota, suggesting that part of their anti-aging effects may be mediated through microbiome-related mechanisms and associated biomarkers. For example, it has been shown that aspirin treatment in mice increases the abundance of protective bacteria such as *Bifidobacterium* and *Lactobacillus*, thereby altering the composition of the mouse gut microbiome (Ref. 52). In the case of metformin, a drug widely used for the treatment of T2DM, several clinical studies have revealed an increase in the proportion of the bacterium *A. muciniphila* in patients treated with metformin, along with enhanced production of SCFAs (Ref. 53). In addition, recent clinical studies have demonstrated the potential of metformin to change the methylation pattern of the DNA through inhibition of DNA methyltransferase activity, leading to gene specific hypomethylation, particularly in the promoter regions of genes involved in longevity, anti-inflammatory responses and tumour suppression, thus facilitating their transcriptional activation (Ref. 54). On the other hand, hypermethylation of genes associated with inflammation and oncogenesis has also been reported in patients with T2DM (Ref. 55).

## Platelets as a source of circulating biomarkers in the aging process

Platelets, small anucleate cells derived from megakaryocytes, have long been recognized for their essential function in haemostasis. However, emerging evidence have expanded this view, highlighting their involvement in a range of biological processes, including inflammation and innate immunity. Due to their short lifespan, platelets are highly sensitive to systemic physiological fluctuations, positioning them as valuable indicators of cellular alterations associated with biological aging (Ref. 56). Platelets circulate with a repertoire of bioactive proteins – such as vascular endothelial growth factor (VEGF), thrombospondin-1 (THBS1) and platelet factor 4 (PF4), which can be selectively taken up from the blood. Several clinical studies have correlated the levels of these proteins with cancer severity as they can contribute to the inflammatory microenvironment (Ref. 57). Notably, the mechanisms through which platelets promote tumour progression may also be relevant to aging, particularly those involving dysregulated angiogenesis and inflammation. For instance, in the context of aging, high levels of PF4 have been detected in platelet-rich plasma from young, healthy individuals. Moreover, systemic administration of PF4 in mice has been shown to rejuvenate peripheral immune function and reduce neuroinflammation (Ref. 58). In addition to their roles in haemostasis and inflammation, platelets are metabolically active cells, making them sensitive indicators of redox balance and oxidative changes. Recent studies have demonstrated that the redox phenotype of platelets exhibits a dynamic, age-dependent profile: while oxidative stress and apoptosis markers increase up to approximately 79 years of age, individuals older than this display an adaptative upregulation of antioxidant mechanisms, resulting in a healthier platelet phenotype (Ref. 59). Furthermore, several studies have shown that mitochondrial function in platelets declines with age. These mitochondrial parameters, such as the maximal oxygen consumption rate, can be reliably measured in intact platelets, supporting their potential as robust and accessible biomarkers of mitochondrial aging (Ref. 60).

## Impact of age-associated diseases on circulating biomarkers

Aging is accompanied by complex physiological changes that influence the expression and reliability of circulating biomarkers. However, the presence of age-associated diseases such as cardiovascular disease (CVD), neurodegenerative disorders and metabolic syndromes can significantly modulate biomarker profiles, making it difficult to distinguish between changes driven by biological aging and those caused by pathological conditions, as these disorders often share molecular mechanisms with aging itself. Therefore, to accurately interpret biomarker data, it is essential to differentiate between healthy elderly individuals and those with clinically diagnosed age-related conditions. In this regard, several studies have demonstrated distinct expression patterns of key biomarkers when comparing these two cohorts (Ref. 61). For instance, IL6 is one of the most recognized sensitive biomarker of vascular aging and low-grade inflammation. Emerging evidence have shown that IL6 levels tend to increase by approximately 0.05 pg/mL per year in healthy individuals. However, in patients with CVD, IL6 levels are significantly higher and have been strongly correlated with increased cardiovascular morbidity and mortality (Ref. 62). In addition, high sensitivity C-reactive protein (hs-CRP) is a well-established biomarker of cardiovascular risk and low-grade inflammation, making it particularly valuable for assessing chronic diseases associated with aging. Indeed, several studies have demonstrated that hs-CRP levels increase with advancing age in elderly individuals and it has been shown that hs-CRP levels remain significantly lower in healthy aging adults compared to those affected by age-related pathologies (Ref. 63), reflecting meaningful differences between these two cohorts.

## Conclusions and outlook

There is no single biomarker with sufficient potential to monitor and control aging. The use of different biomarkers is required as a strategy to control aging and the pathologies associated with it. There are biomarkers currently implemented in the clinical laboratory, such as the CD4/CD8 ratio or MDA levels. However, it is necessary to continue to research new biomarkers to implement them in clinical practice in the near future. Protein CD38 level expression, 20S proteasome complex, bacteria *F. prausnitzii* and *Akkermansia muciniphila* and circulating isoform klotho quantification represent a novel strategy that must be further explored to control the progression of aging. In addition, recent evidence suggests that glycans derived from IgG glycosylation are useful biomarkers for monitoring the aging process given their close association with chronic low-grade inflammation. Moreover, platelets are emerging as a promising biomarker reflecting aging by capturing both mitochondrial dysfunction and compensatory antioxidant responses. Their use offers a minimally invasive approach to monitor mitochondrial health and oxidative stress since they can be easily isolated from peripheral blood.

Finally, it has been shown that certain drugs can modulate specific aging biomarkers; for instance, aspirin treatment increases the abundance of beneficial gut bacteria and metformin can influence DNA methylation patterns, thereby promoting the expression of anti-inflammatory pathways. Therefore, these biomarkers and pharmacological interventions represent some of the most promising advances in the field of aging identified in this revision.
